# Celebrating 50 Years of Nationwide Newborn Screening in Hungary—Review, Current Situation, and Future Directions

**DOI:** 10.3390/ijns11040099

**Published:** 2025-10-27

**Authors:** Péter Monostori, Ildikó Szatmári, Ákos Baráth, János Bókay, Marianna Csenki, Zsolt Galla, Balázs Gellén, Nóra Grecsó, Eszter Gyüre, Zita Halász, Krisztina Hegedűs, Judit Kincs, Erika Kiss, Magdolna Kósa, István Lénárt, Andrea Pálmay, Gábor Rácz, Hajnalka Szabó, Léna Szabó, Viktória Tőkési, Andrea Xue, Petra Zsidegh, Attila József Szabó, Csaba Bereczki

**Affiliations:** 1 Metabolic and Newborn Screening Laboratory, Department of Pediatrics, University of Szeged, Korányi Fasor 14-15, H-6720 Szeged, Hungary; a.barath@gmail.com (Á.B.); galla.zsolt@med.u-szeged.hu (Z.G.); gellen.balazs@med.u-szeged.hu (B.G.); grecso.nora.ildiko@med.u-szeged.hu (N.G.); gyure.eszter@med.u-szeged.hu (E.G.); kosa.magdolna@med.u-szeged.hu (M.K.); lenart.istvan@med.u-szeged.hu (I.L.); racz.gabor@med.u-szeged.hu (G.R.); szabo.hajnalka@med.u-szeged.hu (H.S.); bereczki.csaba@med.u-szeged.hu (C.B.); 2 Metabolic Screening and Diagnostic Center, Department of Pediatrics, Semmelweis University, Bókay János u. 53-54, H-1083 Budapest, Hungary; szatmari.ildiko@semmelweis.hu (I.S.); bokay.janos@semmelweis.hu (J.B.); halasz.zita@semmelweis.hu (Z.H.); hegedus.krisztina1@semmelweis.hu (K.H.); kincs.judit@semmelweis.hu (J.K.); kiss.erika@semmelweis.hu (E.K.); palmay.andrea@semmelweis.hu (A.P.); szabo.lena@semmelweis.hu (L.S.); tokesi.viktoria@semmelweis.hu (V.T.); xue.andrea@semmelweis.hu (A.X.); zsidegh.petra@semmelweis.hu (P.Z.); szabo.attila@semmelweis.hu (A.J.S.); 3 Department of Medical Genetics, University of Szeged, Szőkefalvi-Nagy Béla u. 4/B, H-6720 Szeged, Hungary; csenki.marianna@med.u-szeged.hu

**Keywords:** diagnostics, inborn errors of metabolism, inherited disorders, mass spectrometry, newborn screening

## Abstract

Newborn screening (NBS), one of the most important public health care prevention programs, aims at the early identification of asymptomatic newborns at increased risk for inherited disorders, facilitating timely intervention to reduce morbidity and mortality. NBS in Hungary is celebrating the 50th anniversary of the nationwide implementation of screening for phenylketonuria and galactosemia, as well as the 40th anniversary of congenital hypothyroidism screening. The present paper reviews the early years, the present situation, and future perspectives for the Hungarian NBS program. Today, screening for 27 disorders (opt-out) plus spinal muscular atrophy (opt-in) is supported by two centralized and well-equipped laboratories in Budapest and Szeged, in-depth laboratory knowledge, a robust follow-up system, and governmental financial support. Since 1975, 3,289 patients have been confirmed with a screened condition from over 5.6 million newborns screened. The 50-year anniversary of the Hungarian NBS program highlights the dedication of both past and current professionals, ongoing advancements in analytical methods and laboratory information management systems, and alignment with international standards. The equitable provision of screening services continues to be prioritized for all newborns nationwide and within the broader Euro-regional context.

## 1. Introduction

Screening is the systematic examination of an asymptomatic population for a given disorder, with the aim of identifying individuals at increased risk. The most important screening program from a public health care perspective is newborn screening (NBS), which allows for the detection of several inherited disorders, including inborn errors of metabolism (IEMs), from a blood sample dried on filter paper (dried blood spot; DBS) taken a few days after birth [1,2,3]. The significance of NBS lies in the fact that the early detection of affected newborns provides an opportunity for a timely intervention to avoid irreversible damage and to reduce morbidity and mortality. Over the decades, NBS has evolved from testing for a single disorder, phenylketonuria (PKU), to encompassing large panels of conditions, made possible by advances in medical technology and the understanding of rare disorders. Today, NBS represents a cornerstone of preventive pediatric care in many countries [1,2].

The origins of NBS date back to the early 1960s, when American microbiologist Dr. Robert Guthrie and his assistant Ada Susi developed a simple and reliable bacterial inhibition assay to detect PKU from DBS [4]. The Guthrie test, first implemented as a statewide public health program in Massachusetts in 1963, allowed for mass screening of asymptomatic newborns, which laid the foundation for population-wide metabolic disorder detection [5,6]. The success of PKU screening prompted interest in expanding this concept to other rare and treatable conditions. Pilot programs began across the United States, in Canada (the first country in which a provincial NBS pilot for congenital hypothyroidism (CH) was implemented in 1974) [7], and in several other countries, first with traditional techniques that were later complemented with methods using tandem mass spectrometry (MS/MS) and molecular biology [8,9]. Many of these developments have been reviewed retrospectively on the 50-year anniversary of the implementation of NBS for various disorders [10,11,12,13].

In Europe, NBS began to take shape during the late 1960s and early 1970s [1,2]. The European context, however, presented unique challenges, including differing health care systems, funding mechanisms, and political structures that influenced the pace and scope of implementation. Over time, collaborations, such as those facilitated by the European Society for Phenylketonuria and Allied Disorders Treated as Phenylketonuria (ESPKU), helped harmonize practices and standards. By the 2000s, most European countries had adopted some form of NBS, though the composition of screening panels varied widely. In July 2009, the European Commission launched a tender on NBS with the aim “to report on current practices of laboratory testing, form a network of experts and provide guidance on how to further implement NBS screening in a responsible way” [14]. Recommendations on NBS from this project were published in the “Newborn screening in Europe—Expert Opinion document” in 2011 and in a summarizing policy paper in 2014 [14,15]. Recently, general guidelines for NBS were published by the International Society for Neonatal Screening (ISNS) [16]. Today, the European situation continues to evolve, with ongoing efforts toward standardization and expansion under initiatives such as Screen4Rare and the European Reference Networks (ERNs) [17]. On the occasion of the 50th anniversary of the initiation of nationwide metabolic screening in Hungary, we review the initial years, the current situation, and the future directions of the Hungarian NBS program.

## 2. Newborn Screening in Hungary: Implementation, Early Years, and the Era of Traditional Methods (1968–2007)

Newborn screening in Hungary began with the formation of the NBS laboratory in the Department of Pediatrics of the University of Szeged on 1 January 1968. Effective work started on 12 February, initially covering the city and its surroundings [18,19]. Gradually, NBS was extended to nine counties plus an additional seven large cities in other counties sending their samples to Szeged. To facilitate the inclusion of NBS, detailed information describing PKU, the role of prevention, and institutional tasks and responsibilities was provided to all birth institutions. At this stage, blood sampling was performed on days five to six in term newborns and on day fourteen in preterms. DBS samples were shipped to the NBS center once a week. The effectiveness of the screening program was controlled by monitoring the number of DBS collection cards sent and received, comparing them with the number of births [18]. In a period of four and a half years, 13 newborns with classical PKU were detected in the area covered. Unfortunately, in an additional five children, PKU was diagnosed at a later age with clinical symptoms, because blood sampling had not been performed despite the recommendations. This observation further emphasized the importance of the widest possible coverage of newborns [18].

By 1972, NBS in Hungary had become so widespread that the screening center in Szeged was complemented by a second NBS laboratory in Budapest (location: St. John Hospital 1972–1986, the National Institute of Infant and Pediatric Medicine 1986–1994, Buda Children’s Hospital 1994–2007). By 1974, countrywide coverage of NBS was achieved, with newborns in eight counties plus the capital city screened by the Budapest center and newborns in the remaining eleven counties by the Szeged center, each responsible for an approximately equal number of newborns [18,19]. Beginning in 1975, screening for galactosemia was included with PKU [18,19,20].

A Ministry of Health Decree (Decree No. 5/1975) on 28 May 1975 initiated nationwide metabolic screening [21]. This decree was a cornerstone for the 50th anniversary of the Hungarian NBS program that is being celebrated in 2025. NBS was continued in the centers in Budapest and Szeged (both are still responsible for NBS today), with PKU and galactosemia as the first disorders to be screened. The centralized health care system facilitated uniform implementation and data collection, contributing to early successes in identifying affected newborns and initiating dietary treatment. Using an opt-out approach, the participation rates were over 95% from the beginning, with further improvement within a few years.

The timing of blood sampling was shifted forward from day four to day six in term newborns and the DBS samples were shipped to the NBS laboratories more frequently, twice a week. Both screening tests were based on the slightly modified Guthrie bacterial inhibition method. NBS specimens were autoclaved to denature the proteins and prevent hemoglobin from diffusing into the agar. Comparisons with blood spots spiked with the respective analyte allowed the generation of semi-quantitative test results. Positive screening results for PKU were confirmed by serum and urine examinations. Positive cases for galactosemia were confirmed by Beutler and Weidemann tests as second tiers, as well as determination of the galactose-1-phosphate uridyltransferase (GALT) enzyme activity and the galactose level in erythrocytes [18,19,20]. The Guthrie bacterial inhibition method continued to be used until 2007 for PKU (until the introduction of the MS/MS technique) and until 2004 for galactosemia, respectively.

In 1985, NBS was expanded to include CH, with pilot studies in Győr-Sopron county between 1979 and 1983 [22], from 1982 in Budapest [23], and from 1983 in Szeged [24]. Even if thyroxin had previously been investigated as a potential biomarker, routine NBS used thyroid-stimulating hormone from the beginning using non-autoclaved specimens. In 1993, the radioimmunoassay technique applied in the first years was changed to a dissociation-enhanced lanthanide fluorescence immunoassay (DELFIA™) kit.

According to a review article using NBS data from counties covered by the Szeged lab, 148 patients with PKU and 40 with hyperphenylalaninemia were identified between 1968 and 1988, plus 11 cases with GALT and 5 with galactokinase deficiency, respectively. Between 1985 and 1988, 37 CH patients were also diagnosed. There was a single false-negative case in PKU screening and no known missed cases for galactosemia or CH. A detailed calculation of the costs revealed that NBS was highly cost-effective [19].

**Figure 1:**
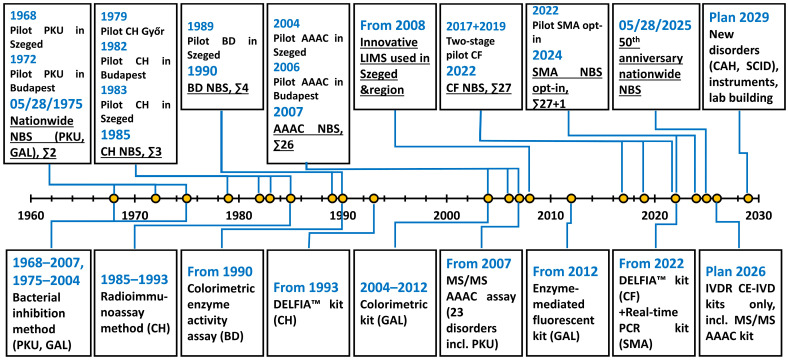
Timeline of major events of the Hungarian newborn screening program. AAAC: amino acid-acylcarnitine; BD: biotinidase deficiency; CAH: congenital adrenal hyperplasia; CE-IVD: Conformité Européenne (European Conformity)—In Vitro Diagnostics; CF: cystic fibrosis; CH: congenital hypothyroidism; DELFIA™: dissociation-enhanced lanthanide fluorescence immunoassay; GAL: galactosemia; IVDR: Regulation No. 2017/746 of the European Union on in vitro diagnostic medical devices; LIMS: laboratory information management system; MS/MS: tandem mass spectrometry; NBS: newborn screening; PCR: polymerase chain reaction; PKU: phenylketonuria; SCID: severe combined immunodeficiency; SMA: spinal muscular atrophy.

In 1989, NBS for biotinidase deficiency was initiated in 5 of the 11 counties shipping to Szeged, but was suspended after five months due to technical problems (unavailability of a reagent). Screening was continued nationwide from 1 May 1990, becoming the fourth disorder in the panel. An in-house colorimetric enzyme activity assay in DBS was used; confirmation was performed on serum samples [25,26]. Major events marking the development of the Hungarian NBS program are summarized in Figure 1.

The comprehensive regulatory basis for NBS in Hungary was issued on 18 December 1997 with Decree No. 51/1997 of the Ministry of Welfare [27]. Even though the Decree is still in effect with the original numbering, several significant amendments have been made since its first issuing to respond to the changing priorities and progression in NBS internationally and nationally [27].

## 3. Extended Newborn Screening: Introduction of Tandem Mass Spectrometry and Further Developments (2007–2020)

Pilot screening studies for amino acids-acylcarnitines (AAACs) and selective AAAC measurements were performed from 2004 in Szeged and from 2006 in Budapest. Expansion of the Hungarian screening panel from four conditions to twenty-six began on 1 October 2007, with the implementation of an MS/MS multiplex assay for AAAC. Since 2007, 23 disorders, including fatty acid oxidation defects, organic acidurias, and disorders of the amino acid metabolism (such as PKU), have been screened by the means of MS/MS. From 2007 to 2020, the other three were screened using traditional assays [a DELFIA™ kit for CH; an in-house colorimetric enzyme activity assay for biotinidase deficiency; and a colorimetric kit for galactosemia (since 2004), which was switched to an enzyme-mediated fluorescent kit in 2012 (both detected total galactose)] [28,29,30,31]. (Figure 1). Of the two NBS centers, the one in Szeged continued to operate in the Department of Pediatrics at the University of Szeged, while in Budapest, the 1st Department of Pediatrics at the Semmelweis University was entrusted with this role. The timing of blood sampling was shifted to 48–72 h, with DBS specimens shipped to the NBS laboratories by priority mail on all working days (unfortunately, delayed shipping still occurs occasionally). Participation rates have been constantly over 99% using the opt-out approach.

The introduction of MS/MS into NBS in the 1990s replaced the “one test, one disorder” strategy with a new strategy of “one test, many disorders.” To facilitate this transition, laboratories in other countries (Austria and Canada, among others) were visited to gain technical knowledge. Over time, new methodologies were implemented in Hungarian laboratories that took advantage of international guidelines and local experiences. This included participation in the Region 4 Stork (R4S) Collaborative Project [32,33] and its successor [Collaborative Laboratory Integrated Reports (CLIR) (https://clir.mayo.edu)] [34] and utilization of guidelines from the Clinical and Laboratory Standards Institute (CLSI) [35,36,37,38,39]. A new laboratory information management systems (LIMS) was implemented to cope with the greater number of disorders and analytes. Both NBS centers developed and used their own dedicated software for both epidemiological data and analytical result management, including patient registration, specimen testing, result follow-up, and quality management. Protocols for clinical evaluation and patient follow-up were also updated regularly.

An innovative remote data entry system was added to the LIMS in Szeged in 2008 to replace the hand-written leaflet attached to the screening card. Individual birth institutions began registering all patient data locally themselves, followed by electronic transfer to the screening laboratory via secure internet clients. An individual bar code is attached to the collection card by the sender, together with the name of the child and the birth date for identification. A part of the bar code specific to the given birth institution decreases the possibility of accidental sample mix-up and allows the birth institutions to remotely track the status of their DBS specimens. This strategy has also improved data security, since sensitive personal data are transferred within a secure system. Upon receipt at the laboratory, the bar code is read and the name and birth date are checked to ensure the proper data and specimen linkage. This approach has shortened the specimen check-in time at the screening laboratory, increased throughput, and decreased the overall laboratory administrative workload. Moreover, specimens delayed or lost during shipping can also be easily recognized and the sender quickly notified for retesting.

Quality assurance of the two NBS laboratories includes participation in international programs and the daily use of quality control (QC) samples. Both centers have been members of the Newborn Screening Quality Assurance Program (NSQAP) provided by the Centers for Disease Control and Prevention (CDC) [29,40] from the beginning of their screening efforts. This includes participation in QC and Proficiency Testing (PT) schemes to evaluate the quantitative and qualitative reliabilities of their MS/MS and traditional first- and second-tier tests.

Diagnostic assays that are not covered by the CDC NSQAP schemes are quality-checked by using samples from the European Research Network for the Evaluation and Improvement of Screening, Diagnosis and Treatment of Inherited Disorders of Metabolism (ERNDIM) [41]. Both laboratories participate in the Qualitative Organic Acids in Urine (QLOU) and Pterins in Urine (PTU) programs. Further schemes include the Special Assays in Serum (SAS) in Budapest, and the Neurotransmitters in Cerebrospinal Fluid (NCSF) and the Special Assays in DBS (SADB) in Szeged.

## 4. Improvements Since 2020 and the Current Situation of Newborn Screening in Hungary

Hungary has long been among the countries that screen for the greatest number of inborn disorders in Europe [1,2]. The screening panel has further been extended from January 01, 2022, to include 27 disorders, with the implementation of cystic fibrosis (CF) NBS [42], preceded by a two-stage pilot study in 2017 and 2019 [43]. A biochemical screening protocol using immunoreactive trypsinogen (IRT) and pancreatitis-associated protein (PAP) has been implemented using an IRT1/IRT*PAP/IRT2 three-tier approach, complemented with a Safety Net strategy. A sweat chloride test and mutation analysis plus sequencing are used for confirmation of screening positive cases [43,44,45].

Recently, as the first disorder to be screened by using molecular biological tests, a pilot study for the NBS of spinal muscular atrophy (SMA) was run between 1 November 2022 and 31 December 2023 using a real-time polymerase chain reaction kit and performed in the same two centers that are responsible for regular NBS [46,47,48]. As previous screenings solely utilized biochemical tests, this project required that the infrastructure for high-throughput genetic screening was established in the two laboratories, supported by a grant from the Ministry of Human Resources. The LIMS software was also updated to allow for the inclusion of SMA, together with further improvements in safety and easier handling. For quality assurance, we participated in the SMAPT scheme from the CDC [49]. In contrast with regular NBS using an opt-out strategy, participation in the SMA pilot screening was voluntary, requiring a written parental informed consent (opt-in). Following numerous information campaigns, the participation rate increased continuously, reaching 85% by the end of 2023. Altogether, nine patients were detected during the pilot study; all of them were confirmed to have the disease and received appropriate therapy [46]. Since 2024, SMA screening has been continued with the same conditions and quality assurance, with a participation rate of over 90%. It is planned that SMA will be included in the regular opt-out screening panel in the near future [46] (Figure 1). A summary of current first-tier methodologies, primary analytes, and screened disorders is provided in Table 1.

Both NBS laboratories purchased new MS/MS instruments in 2020, generously supported by a Ministry grant. Previous AAAC assays were transferred to the new instruments. As an additional improvement in Szeged, succinylacetone, the diagnostic marker of Tyrosinemia Type I, was added to the first-tier AAAC test (previously measured in Hungary as a separate second-tier assay). Great emphasis has been placed on maintaining high-quality standards in the Hungarian NBS program. The two laboratories monitor certain quality indicators regularly and update their own processes if needed. Indicators include, for example, the number and rate of unsatisfactory specimens, unscreened newborns, newborns lost to follow-up, timeliness (collection, transport, and reporting), success rate in external QC schemes, missed cases, and positive predictive value [50,51]. Most indicators have remained constant in the last decade, owing to strict regulations from the NBS Decree and the opt-out system [27]. Second-tier assays are also applied (such as PAP in CF NBS) and additional second-tier assays are considered to reduce false-positive rates further. The transport time of the samples from the birth institutions need improvement (late arrivals are often due to delays in sending the specimen and not the transport itself). The inclusion of CF into the screening panel has resulted in an increase in the number of false positives [43]. One reason is that the specificity of IRT is relatively low when compared with most biomarkers used in NBS [44,45]. In addition, new screenings often perform suboptimally in the first period after their implementation [52], as also seen in Hungary. In contrast, there have been no false negatives or false positives in SMA NBS so far. A summary of the quality indicators can be found in Table 2.

We generally use the following strategy when NBS for a new disorder is to be implemented or when a switch to a new analytical assay is planned. Prior to inclusion of an additional disorder into the screening panel, a nationwide pilot study is performed. In general, anonymized, non-selected NBS samples left over from regular NBS are analyzed with the new assay (approx. 5000 samples per NBS laboratory), together with known patient samples or abnormal specimens from the CDC or ERNDIM. Similarly, if an existing analytical method is to be switched to a new assay, the two methods are run in parallel on the same normal and abnormal samples (approx. 5000). It is important to analyze a large number of unselected specimens (i.e., not only term newborns with completely normal results but also newborns on therapy, preterms, etc.) to have percentiles that better match the “real-world” population to be screened.

The obtained population-specific (and, thus, laboratory-specific) percentiles are used as cutoffs in the first 6–12 months of regular screening. For new disorders with no previous experience, a slightly lower cutoff may be chosen in the beginning. Even if this may lead to a suboptimal false-positive rate in the first period, the risk of having false negatives can be lowered. Later, cutoffs are refined based on experience from regular NBS, aiming to avoid false negatives while keeping the number of false positives as low as possible. Regular formal and informal discussions between the two NBS laboratories enable a better exchange of experience.

In the 50-year history of nationwide NBS in Hungary, a total of 5,624,767 newborns have been tested. In this period, 3289 patients have been detected, yielding an overall incidence of 1:1710. In the past ten years, 891,923 newborns have been screened, with 1047 confirmed patients (incidence 1:852) (Table 3).

In addition to their responsibilities in NBS, the two Hungarian NBS centers have been making continuous efforts to further respond to the needs of those in the community who live with rare disorders. Recent advancements include an improved high-performance liquid chromatography–MS/MS method for the determination of underivatized amino acids from human serum samples [53], a study on the metabolic control in children with PKU during the COVID-19 pandemic [54], and a paper on the impact of a maternal vitamin B12 deficiency in NBS [55]. Publications on second-tier tests include the identification of various interferents of methylmalonic acid in NBS and clinical diagnostics [56] and the development of a second-tier test for CAH NBS [57]. Moreover, a multiplex and extendable ultra-high-performance liquid chromatography (UHPLC)–MS/MS assay has also been developed and validated for a wide range of neurometabolites and associated biomarkers [58]. This versatile method has been applied in diagnostics and multiple research studies [59,60]. Several recent and ongoing studies include(d) collaboration activities with an increasing number of partners, both internationally (examples are the metabolic centers of Heidelberg, Vienna, and Amsterdam and the biomedical centers of Uppsala and Fukuoka) and nationally (e.g., ELI-ALPS Research Institute, HUN-REN Biological Research Center, DNT Southern Great Plain Neurobiological Knowledge Center, HUN-REN Research Center for Natural Sciences).

## 5. Future Plans and Perspectives

The plans for future improvements in the Hungarian NBS system can be grouped into the following two categories: 1. extension of the screening panel and 2. infrastructural and regulatory advancements.

### 5.1. Extension of the Screening Panel

In view of international recommendations [15,61,62], further extension of the Hungarian screening panel is being considered. Some disorders could be incorporated with relatively low financial investment, as they could be fitted into assays already in use. As an example, severe combined immunodeficiency (SCID) could be detected with the same multiplex manufacturer kit that is used for SMA NBS. Guanidinoacetate methyltransferase (GAMT) deficiency and aromatic L-amino acid decarboxylase (AADC) deficiency could be incorporated in the existing AAAC assays by adding the mass transitions of guanidinoacetate, creatine, and creatinine for GAMT and 3-o-methyldopa for AADC, respectively. As this requires no changes in the sample preparation steps and the measurement settings of the original analytes and internal standards already present in the assay can be used, a complete revalidation of the established AAAC assay may not be necessary for its original analytes according to Article (16) (c) of the Regulation No. 2017/746 of the European Union on in vitro diagnostic medical devices (IVDRs), only for the additional metabolites [63]. SCID and GAMT are among the core conditions of the Recommended Uniform Screening Panel (RUSP) [62], and quality assurance for their screening is already available at the CDC NSQAP.

Congenital adrenal hyperplasia (CAH) is another disorder that could additionally be screened in Hungary. CAH has the highest incidence among inborn errors of steroid metabolism (approximately 1:10,000) [64]. CAH is on the list of disorders recommended as the first to be screened by the “Newborn screening in Europe—Expert Opinion document” in 2011 [15], has been included in the RUSP for decades [61,62], and is screened in a large number of countries [1,2,64]. Unfortunately, this is still not the case in Hungary, even if already, a 1988 review stated that “the laboratory conditions for implementing CAH screening are available” [19]. An elevation in 17-hydroxyprogesterone, detected by the means of immunoassays, is a sensitive biomarker of CAH [64]. The relatively low specificity of 17-hydroxyprogesterone can be markedly improved by second-tier testing [64], for which a high-sensitivity UHPLC-MS/MS instrument for each NBS center needs to be purchased. On the other hand, this procurement could financially be counterbalanced by the relatively low costs of CAH therapy [65] when compared with many screenable disorders, particularly SMA. Further savings are to be expected, such as life savings in salt-wasting forms of CAH when detected early via NBS, as well as lower therapeutic costs and improved quality of life in simple-virilizing cases with symptoms related to sexual development [64].

From the group of lysosomal storage disorders, the RUSP recommends screening for glycogen storage disease Type II (Pompe), infantile Krabbe disease, and mucopolysaccharidoses Type I and Type II [61,62]. The former three disorders are included in an available MS/MS kit, which could facilitate easier method implementation. Using the above kit as a selective assay, enzyme activity measurements using DBS for six lysosomal storage disorders (Pompe, Krabbe, Fabry, Gaucher, mucopolysaccharidosis I, and Niemann-Pick A/B) are available in the Budapest center. In the Szeged center, selective assays using traditional methods on whole blood are currently used to detect Pompe, Fabry, and Gaucher diseases, mucopolysaccharidoses I and IV-B, gangliosidoses GM1 and GM2 (Tay–Sachs, Sandhoff), and metachromatic leukodystrophy, as well as oligosaccharidoses alpha-mannosidosis, and fukosidosis.

X-linked adrenoleukodystrophy (X-ALD) screening would also be feasible through detecting elevated levels of C26:0-lysophosphatidylcholine in the AAAC assay. Unfortunately, this biomarker degrades during derivatization with butanolic HCl, a technique currently utilized by both Hungarian NBS centers. Nevertheless, inclusion of X-ALD would still be possible if the laboratories switched to non-derivatized assays. A transition to non-derivatized manufacturer AAAC kits will also be required in the near future for compliance with the IVDR Regulation (detailed in the following section).

### 5.2. Infrastructural and Regulatory Advancements

Both Hungarian NBS laboratories operate in dedicated buildings in the immediate vicinity of the clinical sections of the Departments of Pediatrics, which facilitates an efficient exchange between clinicians and laboratory scientists in terms of samples and information. The buildings were modernized some years ago to meet the needs of the laboratories. However, the space is becoming small due to constant increases in workload and regulatory requirements. In spite of careful optimization of the analytical processes, an enlargement of the laboratory space will very likely be necessary in the future.

The instruments applied in the AAAC and the SMA first-tier assays are relatively modern due to recent purchases. Procurement of new devices for these first tiers is expected to be necessary in three to five years. However, some of the other instruments are older and will probably have to be replaced soon. The increasing demand for second-tier tests and diagnostic assays for further IEMs will require that a high-sensitivity UHPLC-MS/MS is purchased in both laboratories (which could also be used for a possible implementation of CAH screening).

As concerns upcoming regulatory changes, the Regulation No. 2017/746 of the European Union on in vitro diagnostic medical devices (IVDRs) is of fundamental importance for screening and diagnostic laboratories in EU member countries [63]. According to Article 5 (5), most laboratory developed tests will have to be switched to CE-IVD-certified manufacturer kits. Although the Hungarian NBS program is already using some kits, others, including the AAAC method, need to be transferred. Even if the necessary instrumentation and knowledge for the switch are available in two NBS laboratories, the financial resources are lacking. The current governmental reimbursement would only cover a fraction of the future costs of the screening centers, as CE-IVD-certified manufacturer kits are far more expensive than the current laboratory developed tests. In Hungary, NBS has to be performed automatically for all newborns, in agreement with the Governmental Decree No. 51/1997 [27]. According to Annex VIII, 2.3 (Rule 3) (m) of the IVDR [63], devices used for NBS are classified as class C with an adaptation deadline of 26 May 2026. Therefore, the financial reimbursement of the NBS laboratory measurements will need to be increased in the near future to allow for conformity with the expectations set by both the national Decree and the IVDR Regulation of the European Union [27,63].

## 6. Conclusions

In 2025, the Hungarian NBS program is celebrating the 50th anniversary of its nationwide implementation. On this occasion, the present paper reviews both the advances and the challenges of its beginnings, the present situation, and future perspectives. Since 1975 [21], the number of screened disorders has substantially increased and the analytical technologies and therapeutic protocols have continuously been improved. Today, the Hungarian NBS program includes 27 + 1 conditions [27,29,42,43,46], supported by two centralized laboratories, robust systems for screening and follow-up, and governmental financial support. In the history of our nationwide NBS, more than 3200 patients have been detected from over 5.6 million newborns. Looking ahead, the Hungarian NBS program is ready to face approaching challenges such as further extension of the screening panel and the implementation of the IVDR Regulation, including a transfer to further CE-IVD-certified manufacturer kits [63], requiring governmental approval for increased financing.

The 50th anniversary of the Hungarian NBS program stands as a testament to the commitment of all our past and present staff, continuous professional development, and adherence to international best practices. Hungary remains focused on improving diagnostic coverage and the quality of screening, integrating genomic technologies, and ensuring equal access for all newborns in the country, being open to support screening and diagnostics in the Euroregion.

## Figures and Tables

**Table 1:** Summary of current first-tier methodologies, primary analytes, and screened disorders in the Hungarian NBS program.

First-Tier Methods	Primary Analytes	Screened Disorders
MS/MS AAAC	Ala, Arg, Asp, Cit, Glu, Gly, Leu, Met, Orn, Phe, SuAc, Trp, Tyr, Val, C0, C2, C3, C3DC, C4, C4DC, C4OH, C5, C5:1, C5DC, C5OH, C6, C6DC, C8, C10, C10:1, C10:2, C12, C14, C14:1, C14:2, C16, C16:1OH, C16OH, C18, C18:1, C18OH	3MCC, ASL, BKT, CIT-I, CPT-I, CPT-II, CUD, GA-I, HCYS, HMG, IVA, LCHAD, MADD, MCAD, MCD, MMACBL, MSUD, PA, PKU, SCAD, TYR-I, TYR-II, VLCAD
DELFIA™	TSH, IRT	CH, CF
Enzyme-mediated fluorescence	Total galactose	Galactosemia (GALT and galactokinase deficiency)
Colorimetric enzyme activity	Biotinidase activity	BD
Real-time PCR	SMN1 exon 7 homozygous deletion	SMA

3MCC: 3-Methylcrotonyl-CoA carboxylase deficiency; ASL: Argininosuccinate lyase deficiency; BKT: Beta-ketothiolase deficiency; CIT-I: Citrullinemia Type I; CPT-I: Carnitine-palmitoyl transferase deficiency Type I; CPT-II: Carnitine-palmitoyl transferase deficiency Type II; CUD: Carnitine uptake defect; GA-I: Glutaric acidemia Type I; HCYS: Homocystinuria; HMG: 3-Hydroxy-3-methylglutaryl-CoA lyase deficiency; IVA: Isovaleric acidemia; LCHAD: Long-chain hydroxyacyl-CoA dehydrogenase deficiency; MADD: Multiple acyl-CoA dehydrogenase deficiency; MCAD: Medium-chain acyl-CoA dehydrogenase deficiency; MCD: Multiple carboxylase deficiency; MMACBL: Methylmalonic acidemia and cobalamin defects; MSUD: Maple syrup urine disease; PA: Propionic acidemia; PKU: Phenylketonuria; SCAD: Short-chain acyl-CoA dehydrogenase deficiency; TYR-I: Tyrosinemia Type I; TYR-II: Tyrosinemia Type II; VLCAD: Very long-chain acyl-CoA dehydrogenase deficiency; BD: Biotinidase deficiency; CF: Cystic fibrosis; CH: Congenital hypothyroidism; SMA: Spinal muscular atrophy; AAAC: amino acid-acylcarnitine; DELFIA™: dissociation-enhanced lanthanide fluorescence immunoassay; GALT: galactose-1-phosphate uridyltransferase; IRT: immunoreactive trypsinogen; MS/MS: tandem mass spectrometry; PCR: polymerase chain reaction; SMN1: survival of motor neuron 1; TSH: thyroid-stimulating hormone.

**Table 2:** Summary of quality indicators in the Hungarian NBS program (2014–now).

Quality Indicator/Category	Threshold/Goal
Unsatisfactory specimens	<2%
Missing essential information (data fields)	<0.5% (approx. 0% using the LIMS in the Szeged region).
Unscreened newborns	Very low, close to 0% (opt-out strategy).
Lost to follow-up (no final resolution)	<1%
Timeliness (collection)	Main rule: 48–72h, automatically repeated in certain cases (transfusion, preterm newborn, etc.) (fulfilled in >98% of cases).
Timeliness (transport)	Next weekday with priority (fulfilled in >95% of cases).
Timeliness (reporting)	Within 1 week (fulfilled in >95% of cases).
Repeat testing	Typically <1%.
Proficiency testing	Acceptance rate was 100% in the last ten years.
Missed cases	Six false negatives reported in CF, none reported for other disorders so far (excluding late-onset forms).
Parental refusals	Very low, close to 0% (opt-out strategy).
Positive predictive value (PPV)	Disorder-specific: from <15% (CF, under revision) to 100% (SMA).

CF: Cystic fibrosis; LIMS: laboratory information management system; PPV: positive predictive value; SMA: Spinal muscular atrophy.

**Table 3:** Summary of confirmed patients with disorders screened in Hungary (2015–2024).

	2015	2016	2017	2018	2019	2020	2021	2022	2023	2024	Total
3MCC ^(1)^	17	7	4	4	8	5	7	5	11	4	72
ASL	0	0	0	0	0	1	1	0	0	0	2
BKT	0	0	0	0	0	0	0	0	0	0	0
CIT-I	0	0	0	2	1	0	4	0	0	0	7
CPT-I	0	0	0	0	1	0	0	1	2	0	4
CPT-II	0	0	0	0	0	0	0	0	0	0	0
CUD ^(1)^	1	2	2	1	0	0	1	0	1	2	10
GA-I	1	0	0	0	2	0	0	0	0	0	3
HCYS	0	1	1	0	0	0	2	0	1	0	5
HMG	0	0	0	0	1	0	0	0	0	0	1
IVA	0	0	2	0	0	0	0	0	0	0	2
LCHAD	0	2	0	0	0	0	0	0	2	0	4
MADD	1	0	0	0	0	1	0	0	0	0	2
MCAD	5	5	5	4	7	4	10	8	8	12	68
MCD	0	0	0	0	0	0	0	0	0	0	0
MMACBL ^(2)^	1	1	0	2	1	1	5	11 ^(3)^	16 ^(3)^	4	42
MSUD	1	0	0	0	1	2	0	0	0	0	4
PA	1	0	1	0	3	0	3	1	0	1	10
PKU ^(4)^	15	16	15	14	18	17	14	16	15	17	157
SCAD	17	6	5	2	4	2	11	11	2	3	63
TYR-I	0	0	1	0	2	1	1	0	0	1	6
TYR-II	0	0	0	0	0	0	0	0	0	0	0
VLCAD	0	1	1	1	0	0	2	0	1	1	7
BD	6	5	2	4	7	8	0	1	4	5	42
CF ^(5)^	–	–	–	–	–	–	–	17	26	16	59 ^(5)^
CH	33	33	32	27	52	37	31	25	23	29	322
GAL ^(6)^	8	12	16	9	8	13	27	16	17	10	136
SMA ^(7)^	–	–	–	–	–	–	–	0	9	10	19 ^(7)^
Total patients	107	91	87	70	116	92	119	112	138	115	1,047
Total births	91,690	93,063	91,577	89,807	89,193	92,338	93,039	88,491	88,225	77,500	891,923

^(1)^ Including maternal cases. ^(2)^ Including cobalamin defects and maternal B12 deficiencies. ^(3)^ All cases were maternal B12 deficiencies. ^(4)^ Including hyperphenylalaninemias, tetrahydrobiopterin (BH4) deficiencies and maternal PKU cases. ^(5)^ Screened since 1 January 2022. ^(6)^ Including Galactose-1-phosphate uridyltransferase and Galactokinase deficiencies. ^(7)^ Screened since 1 November 2022 using an opt-in strategy. 3MCC: 3-Methylcrotonyl-CoA carboxylase deficiency; ASL: Argininosuccinate lyase deficiency; BKT: Beta-ketothiolase deficiency; CIT-I: Citrullinemia Type I; CPT-I: Carnitine-palmitoyl transferase deficiency Type I; CPT-II: Carnitine-palmitoyl transferase deficiency Type II; CUD: Carnitine uptake defect; GA-I: Glutaric acidemia Type I; HCYS: Homocystinuria; HMG: 3-Hydroxy-3-methylglutaryl-CoA lyase deficiency; IVA: Isovaleric acidemia; LCHAD: Long-chain hydroxyacyl-CoA dehydrogenase deficiency; MADD: Multiple acyl-CoA dehydrogenase deficiency; MCAD: Medium-chain acyl-CoA dehydrogenase deficiency; MCD: Multiple carboxylase deficiency; MMACBL: Methylmalonic acidemia and cobalamin defects; MSUD: Maple syrup urine disease; PA: Propionic acidemia; PKU: Phenylketonuria; SCAD: Short-chain acyl-CoA dehydrogenase deficiency; TYR-I: Tyrosinemia Type I; TYR-II: Tyrosinemia Type II; VLCAD: Very long-chain acyl-CoA dehydrogenase deficiency; BD: Biotinidase deficiency; CF: Cystic fibrosis; CH: Congenital hypothyroidism; GAL: Galactosemia; SMA: Spinal muscular atrophy.

## Data Availability

All research data can be found in the Hungarian newborn screening database.

## Abbreviations

The following abbreviations are used in this manuscript:

AAAC	amino acid-acylcarnitine
AADC	aromatic L-amino acid decarboxylase
CAH	congenital adrenal hyperplasia
CDC	Centers for Disease Control and Prevention
CE-IVD	Conformité Européenne (European Conformity)—In Vitro Diagnostics
CF	cystic fibrosis
CH	congenital hypothyroidism
DELFIA™	dissociation-enhanced lanthanide fluorescence immunoassay
DBS	dried blood spot
ERNDIM	European Research Network for the Evaluation and Improvement of Screening, Diagnosis and Treatment of Inherited Disorders of Metabolism
GALT	galactose-1-phosphate uridyltransferase
GAMT	guanidinoacetate methyltransferase
IEM	inborn error of metabolism
IRT	immunoreactive trypsinogen
IVDR	Regulation No. 2017/746 of the European Union on in vitro diagnostic medical devices
LIMS	laboratory information management system
MS/MS	tandem mass spectrometry
NBS	newborn screening
NSQAP	Newborn Screening Quality Assurance Program
PAP	pancreatitis-associated protein
PKU	phenylketonuria
PT	Proficiency Testing
QC	Quality Control
RUSP	Recommended Uniform Screening Panel
SCID	severe combined immunodeficiency
SMA	spinal muscular atrophy
UHPLC	ultra-high-performance liquid chromatography
X-ALD	X-linked adrenoleukodystrophy
